# Booms and Busts in Housing Market and Health Outcomes for Older Americans

**DOI:** 10.1093/geroni/igab012

**Published:** 2021-04-10

**Authors:** Dahai Yue, Ninez A Ponce

**Affiliations:** 1 Department of Health Policy and Management, University of Maryland, College Park, USA; 2 Department of Health Policy and Management, University of California, Los Angeles, USA

**Keywords:** Health, Housing price dynamics, Mortgaged owners, Outright owners, Renters

## Abstract

**Background and Objectives:**

The US housing market has experienced considerable fluctuations over the last decades. This study aimed to investigate the impacts of housing price dynamics on physical health, mental health, and health-related behaviors for older American outright owners, mortgaged owners, and renters.

**Research Design and Methods:**

We drew longitudinal data from the 1992–2016 Health and Retirement Study and merged it to the 5-digit zip code–level Housing Price Index. The analytic sample comprised 34 182 persons and 174 759 person-year observations. We used a fixed-effects model to identify the health impacts of housing price dynamics separately for outright owners, mortgaged owners, and renters.

**Results:**

A 100% increase in Housing Price Index was associated with a 2.81 and 3.50 percentage points (pp) increase in the probability of reporting excellent/very good/good health status for mortgage owners and renters, respectively. It was also related to a lower likelihood of obesity (1.82 pp) for outright owners and a lesser chance of obesity (2.85 pp) and smoking (3.03 pp) for renters. All of these relationships were statistically significant (*p* < .05). Renters also experienced significantly decreased depression scores (−0.24), measured by the Center for Epidemiologic Studies—Depression scale, associated with the same housing price changes.

**Discussion and Implications:**

Housing price dynamics have significant health impacts, and renters are more sensitive to fluctuations in the housing market. Our study rules out the wealth effect as the mechanism through which changes in housing prices affect older adults’ health. Our findings may inform policies to promote older adults’ health by investing in local area amenities and improving socioeconomic conditions.

**Translational Significance:** The US housing market has experienced multiple booms and busts over the past decades, which should have substantial health impacts for older adults. This study leverages temporal and geographical changes in zip code–level housing prices to estimate the health effects of housing price dynamics for middle-aged and older American adults. Understanding these health impacts could inform policymakers to propose targeted inventions to promote older adults’ health during the housing market’s booms and busts.

The US housing market has experienced multiple booms and busts over the past decades. Housing prices peaked in early 2006 and then declined to new lows in 2012. The bursting of the housing bubble followed by the credit crisis was an important cause of the 2007–2009 Great Recession. Since then, housing prices have been climbing at a fast rate again. Fluctuations in housing prices have particular impacts on middle-aged and older adults; about 55% of households are headed by someone aged 50 years or older, and over 70% of them own a home (1). These fluctuations could exhibit health impacts via a direct wealth effect and an indirect effect of amenities and economic opportunities (2–4). Moreover, housing is also an important determinant of health. Many studies have found an association between housing and better physical health, mental health, and longevity (5–8). The relationship is particularly strong for older adults (9).

In addition, housing serves as the primary component of the wealth portfolio for US older adults. Housing price dynamics could have profound impacts on health through changes in wealth, consumption, labor supply, or marital stability (3,10,11), although older adults show little interest in using housing wealth to support their general nonhousing consumption needs (12–14). For home renters, rising housing prices could make it harder for them to buy a house if they wish to and should be associated with higher rental payments. In this way, it could impose detrimental effects on renters’ well-being. On the other hand, housing prices may also capture local economic growth that exhibits beneficial health impacts. A booming housing market might reflect a growing economy with more jobs and a healthy living environment, which would improve health for everyone. As these strands of evidence present no consistent prediction, the net effect of housing price dynamics on health and how it differs for homeowners and renters are thus far from clear.

In this study, we aimed to estimate the health effects of housing price dynamics for middle-aged and older adults by using panel data and individual fixed-effects models that leverage temporal and geographical changes in zip code–level housing prices. We also investigated heterogeneous effects by housing tenure status (eg, outright owners, mortgaged owners, and renters). Our estimates provide new evidence on the impacts of housing prices on older adults’ health, which could help policymakers and researchers propose effective targeted interventions for older adults as housing prices change.

## The Relationship Between Housing Prices and Health

The dramatic fluctuations in the housing market around the Great Recession have provoked extensive literature examining the effects of housing prices on various economic and health outcomes taking advantage of the geographic variation in magnitude and timing of housing price movements. These studies have shown the importance of housing prices on consumption (15–17), labor supply (18,19), local employment (20), and marital stability (11). However, research on the relationship between housing prices and health status is relatively sparse. Changes in housing prices could affect health through a variety of mechanisms that may differ for renters, outright owners, and mortgaged owners.

### Wealth Effect

Much of prior research on health outcomes has focused on the wealth effect mechanism for nonolder adults (those younger than age 65). For home renters, rising housing prices could raise living costs, which would impose detrimental effects on renters’ health status. For homeowners, because rising housing prices would result in wealth gains, individuals (especially nonolder adults) may access those wealth gains through home equity loans, reverse mortgages, and formal or informal borrowing markets. As predicted by standard lifecycle consumption theory, permanent and unanticipated shocks to wealth would lead to an adjustment of consumption of goods and services (19). Because health is considered a normal good (21), wealth gains from rising housing prices would increase individuals’ health-enhancing consumption and investment. We expect that the wealth effect for outright owners differs from that for mortgaged owners. The reasons include that the mortgaged owners have the risk for foreclosure during a busting housing market while anticipating higher mortgage repayments if they plan to upgrade their housing during a booming market (2). However, this is not the case if homeowners consider the wealth gains as transitory (22) or imperfections in capital markets preclude them from accessing gains in housing wealth. It is very likely to be true for older homeowners. Indeed, previous studies have pointed out that older adults have little interest in reverse mortgages that allow them to draw down home equity while staying in the home (12,13). Also, older adults generally do not use housing wealth to support general nonhousing consumption needs; large reductions in home equity were typically precipitated by family shocks such as death or severe illness (14,23). Thus, housing price dynamics are unlikely to affect older adults’ health through a wealth mechanism.

Most studies examined the wealth mechanism for nonolder homeowners by leveraging the variation in housing prices geographically and over time. For example, a study merging county-level house prices to the 1993–2008 British Household Panel Survey reported that rising house prices lower the likelihood of homeowners exhibiting several medical ailments and improve self-reported health, however, with no effect on their psychological health (3). Similar results were found in a study using Australian data; increases in local house prices were associated with a positive effect on outright owners’ physical health but a detrimental impact on both the physical and mental health of renters (2). Yet, studies in the United States are limited and provide inconsistent results. There is evidence that diminished housing wealth from the Great Recession resulted in higher rates of depression for homeowners (mostly nonolder adults), especially for mortgage owners (10). Foreclosure during the recession period was also associated with more hospital visits and emergency room visits and affected already vulnerable populations (24,25). A recent study examined the impact of housing prices on the health of adults of all ages using the 2002–2012 Behavioral Risk Factor Surveillance System (26). It found positive effects on homeowners’ mental health associated with increases in housing prices. It also reported adverse effects on renter’s health and health-related behaviors when housing prices increase, but these effects were not persistent in the long run.

Studies investigating the health impacts of housing price dynamics for older adults are pretty limited. One study found that declines in housing prices were associated with increased utilization of antidepressant prescriptions for the nearly older adults population in the United States (27). But the authors did not report the differential effects for homeowners and renters. Using a sample of 4207 homeowners, born between 1924 and 1960, from the Health and Retirement Study (HRS), Hamoudi and Dowd (28) found a statistically significant lower risk of anxiety for women and better performances on some cognitive measures for homeowners when housing prices increase from 1992 to 2006. However, in another study with the same data for homeowners and additional 713 HRS-respondent renters in the same birth cohorts, the authors found no statistically significant changes in most of the physical health measures associated with increases in housing prices for both homeowners and renters (29).

### Local Amenities and Socioeconomic Prospects

Another mechanism for the relationship between housing prices and health is local area amenities. Improvements in the local living environment (eg, green space, crime, school quality, and commuting time to central business districts) are quickly capitalized into housing prices (30,31) and have health implications for residents. For example, the amount of green space in a neighborhood has significant effects on individuals’ perceived general health, with a stronger relationship for lower socioeconomic groups (32). These environmental variables would affect anyone living in the neighborhood regardless of home tenure status.

The housing market may also reflect local business cycles, such as employment and economic outputs, which exhibit health impacts for both homeowners and renters. Leamer (2007) (33) considers housing prices as an important precursor of the national business cycles or economic development. Several other studies have also shown that rising house prices lead to corporate investment, business expansion, and job creation (20,34). A decline in aggregate housing prices leads to a decrease in the gross domestic product (35). If renters could get higher wages that offset the surged rental payment during a booming housing market, renters would likely see more health benefits. On the other hand, renters are more likely to face the risk of eviction when losing jobs during an economic recession, which would have substantial adverse effects on their health. In addition, a strong housing market may bolster confidence in the economy or foster a “feel-good” factor for many forward-looking individuals as a distinct barometer of economic prospects. If it is the case, an increase in housing prices will result in improved health. For example, Ratcliffe (4) confirms this possibility by showing a positive association between house prices and the mental health of UK homeowners and nonhomeowners, with larger effects for the latter.

In summary, previous theories and the existing evidence do not provide a clear prediction of the relationship between housing prices and health. Assuming wealth effects dominate, we would expect beneficial health effects for homeowners and detrimental consequences for renters, associated with increases in housing prices. Assuming the area amenities and economic prospects dominate, renters should experience at least as many health benefits or detrimental consequences as homeowners. Therefore, how housing price dynamics affect health outcomes is more of an empirical question.

## Method

### Data

The microdata for the analyses were the 1992–2016 HRS. HRS is a longitudinal panel study that surveys a representative sample of individuals aged 51 and older and their spouses in the United States. Starting from 1992 with biennial interviews, HRS collects information about demographics, income, assets, health, and housing in its core files. The HRS housing module contains extensive housing-related information such as homeownership, home value, and mortgages. We extracted those housing-related variables from HRS, then merged them to RAND HRS Longitudinal File 2016 (RAND HRS). RAND HRS is a cleaned version of the HRS core interviews with variables derived and imputed consistently across waves. We used restricted geocoded HRS data with 5-digit zip code identifiers to link Housing Price Index (HPI) and other socioeconomic variables.

We matched into the HRS data the 5-digit zip code–level HPI obtained from the US Federal Housing Finance Agency (FHFA) based on the location the household lived in at the time of the interview. FHFA constructs HPI using repeat mortgage transactions on single-family properties whose mortgages have been purchased or securitized by Fannie Mae and Freddie Mac. It is a weighted, repeat-sales index capturing the movement of single-family house prices holding home quality constant. As such, it avoids the problem of changes in the composition of sales that affect localized average house prices. The year 2000 is the base year for HPI. Therefore, HPI should be interpreted as the cumulative percent change in housing prices relative to 2000.

Additionally, to control for local economic conditions and access to health care, we also matched into HRS a list of area-level socioeconomic variables from 1992 to 2016, including county-level median household income and poverty rates from the Small Area Income and Poverty Estimates Program (36), county-level unemployment rates, and the number of hospital beds from the Areas Health Resources Files (37).

### Measures

To capture respondents’ multiple facets of health status, we included 4 outcomes to measure individuals’ physical health, mental health, and health-related behaviors. For physical health, we used self-reported good health status (excellent/very good/good versus fair/poor). Prior studies have validated that self-reported health is strongly predictive of mortality and objective health outcomes such as functional ability (38,39). The proxy for mental health was depressive symptoms, measured as a count variable by the respondent’s score on an 8-item version of the commonly used Center for Epidemiologic Studies—Depression (CES-D) scale. CES-D scale has been proved to be an effective screening instrument for clinical depression for community-residing older adults (40). The score is the sum of responses to 6 unfavorable indicators (always or much of the time feeling depressed, sad, or lonely, feeling that everything is an effort, feeling unable to get going, or having restless sleep) and 2 reverse-coded favorable indicators (feeling happy and enjoying life). A score of 8 indicates the highest risk of depression.

We used 2 measures available in the HRS data to reflect respondent’s health-related behaviors—obesity defined as body mass index of at least 30 (constructed as weight in kilograms divided by the square of height in meters) and whether or not smoking at the time of interview. We selected smoking and obesity as proxies for health-related behaviors because they are leading causes of mortality in the United States (41,42) and commonly used indicators for health behaviors in the health economics literature (43,44).

Based on the housing-related items collected by HRS, we defined 3 categories of housing tenure status: outright owners (those who are owning or buying their homes without mortgages), mortgaged owners (owning or buying their homes with mortgages), and renters. We excluded those with responses to housing ownership questions as “lives rent-free with relative/employer/friend” or “others,” and those with missing values in the housing ownership question.

We included several individual demographic variables in the analyses: age, sex, whether US born, race (White, Black, and other), Hispanic, educational attainment (less than high school, high school, some college, and college and above), year-specific marital status (married, divorced, widowed, and never married), and year-specific labor force participation status (working, partly retired, disabled, and outside labor force). In addition, we included 2 wealth measures: nonhousing wealth and housing wealth. Housing wealth is defined as the value of primary residence plus the net value of the real estate (not a primary residence). For temporal compatibility, wealth measures are assessed at constant 2016 values using the Consumer Price Index from the US Bureau of Labor Statistics. To reflect fluctuations in local social and economic conditions, we obtained 3 county-level economic indicators (all-age percent in poverty, median household income, and unemployment rates for those aged 16 or older) and one proxy for changes in health care resources (county-level number of hospital beds).

### Statistical Model

Because HRS is a longitudinal panel study, we applied an individual fixed-effects model to accommodate the within-respondents correlation. The identifying assumption is that the geographic variation in the scale and timing of housing price is conditionally exogenous to health. The statistical model is written as follows:


$$\begin{array}{l} \begin{array}{ll} & Health_{izt}=\alpha_{0}+\alpha_{1}MortgagedOwner_{izt}\\ & +\;\alpha_{2}Renter_{izt}+\beta_{1}\ln\left(HPI_{zt}\right)\\ & \times \;\;OutrightOwner_{izt}+\beta_{2}\ln\left(HPI_{zt}\right)\;\\ & \;\times \;MortgagedOwner_{izt}+\beta_{3}\ln\left(HPI_{zt}\right)\\ & \times \;Renter_{izt}+X_{izt}^{{\;}^{\prime }\;}\gamma +H_{ct}^{{\;}^{\prime }\;}\theta +\lambda_{i}+\mu_{ict}+\phi_{t}\\ & +\;S_{it}\times T+{\;\varepsilon \;}_{izt} \end{array} \end{array}$$

Where *OutrightOwner*_*izt*_, *MortgagedOwner*_*izt*_, and *HomeRenter*_*izt*_ are indicator variables, representing the housing tenure status for individual *i* living in an area with zip code *z* in year *t*. *X*_*izt*_ is a vector of individual-level time-varying variables including age, the square of age, marital status, and labor force participation status. *H*_*ct*_ contains county-level time-varying economic conditions (poverty rates, median household income, and unemployment rates) and health resources (number of hospital beds). λ _*i*_ denotes individual fixed effects, μ _*ict*_ captures county-by-year fixed effects, and ϕ _*t*_ is year dummies to hold constant those that vary uniformly across states over time. Given the evidence that historically movements in house prices are driven by local (state- or region-specific) components rather than national shocks (45), we included state-specific linear trends to reflect state-level changes in policies, investment, climate, and other confounding factors. Finally, ε _*izt*_ is the error term. Standard errors were clustered at the individual level.

The interaction terms between HPI and housing ownership allow the effects of house price dynamics to vary by homeownership status. As such, β _1_, β _2_, and β _3_ capture the health impact of housing prices for outright owners, mortgaged owners, and renters, respectively. We used a log-transformed HPI measure to approximate a normal distribution (Supplementary Figure 1).

It is worth noting that we estimated the model using a linear within-individual fixed-effect regression model throughout for 2 reasons. First, linear regression performs better in the presence of interaction terms. A linear model yields consistent estimates for noncontinuous outcomes in panel fixed effects, given large sample size (46,47). It also allows for direct interpretation of the coefficients on interaction terms, which is widely employed in studies analyzing health effects of housing price dynamics with panel data (2–4,29). In contrast, coefficients on interaction terms from nonlinear models are not immediately interpretable (48–50). For example, coefficients on the interaction terms from a logit model represent the natural logarithm of the ratio of 2 odds ratios; the complexity and lack of policy meaning further discourage the odds ratio interpretation (50). More importantly, it is not possible to convert these coefficients to marginal effects (eg, probabilities) from a fixed-effects model; we are unable to make predictions as the fixed effects are conditioned out of the likelihood function (48,50). Second, nonlinear models perform poorly with fixed effects (51–54). Also, in short panels, fixed-effects estimators of nonlinear models can be severely biased due to the incidental parameter problem (55,56).

To assess our results’ robustness, we first explored the extent to which selective migration may bias the estimators by restricting the sample to nonmovers. We then estimated a fixed-effects instrumental variable (FEIV) model using a 2-year prior lagged log HPI as an instrument, as it is less likely that health outcomes can influence past values of residency-area HPI. The FEIV model addresses the concern that HPI may capture the effect of a coincident event or shock. Additionally, as HPI was measured at the zip code level, we show the results from our main specification, adjusting for zip code fixed effects. It is worth noting that we included county, instead of zip code, fixed effects in the main analyses for 2 primary reasons. First, the number of respondents per zip code is pretty small. About 30% of zip codes only include one respondent, and approximately 90% of zip codes have less than 10 individuals. The intraclass correlation within zip codes is less than 0.1. Second, controlling for county fixed effects has more policy implications because most public health policies are made at the county level (57). It is also quite common for individuals to use resources (eg, hospitals, parks, and gyms) across zip codes but uncommon across counties. Third, we investigated the mediation effect of wealth in the relationship between HPI and health outcomes by additionally controlling for wealth in the main regression models (43,58). Fourth, we assessed the importance of county-level economic and health controls by excluding them from the primary model. Finally, we performed subgroup analyses by sex and by whether homeowners own multiple properties.

## Results

The analytic sample comprises 34 182 persons and 174 759 person-year observations for eligible respondents plus his/her spouse from the 1992–2016 HRS. We excluded those with missing values in housing ownership, zip code, HPI, outcomes, or other covariates. Supplementary Figure 2 shows the sample flowchart.

**Figure 1.**
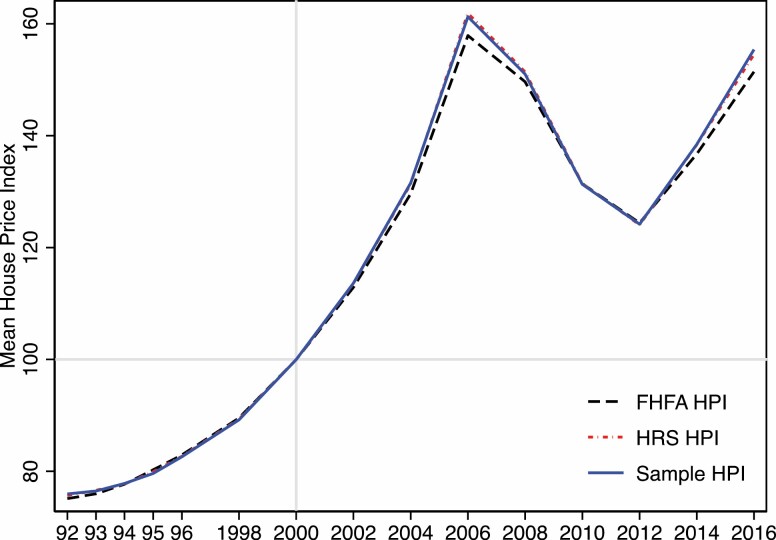
Temporal trends of an average house price index. *Note:* FHFA HPI represents the mean value of Housing Price Index (HPI) calculated based on the Federal Housing Finance Agency (FHFA) database, HRS HPI denotes average HPI using the Health and Retirement Study (HRS) full sample, and Sample HPI indicates the mean HPI based on the HRS analytic sample.

To confirm HRS respondents are not concentrating in areas with unusually high or low housing prices, Figure 1 presents the HPI trends calculated from the FHFA HPI national data set, all HRS respondents, and analytic HRS sample from 1992 to 2016, respectively. These trends display a consistent pattern that housing prices went up before 2006, declined from 2006 to 2012, and are recovering after 2012. For example, the HPI in Los Angeles County of California was 100 in 2000, 246.6 in 2006, 161.4 in 2012, and 228.4 in 2016. Trends in the median level of HPI are similar (Supplementary Figure 3).

Table 1 presents summary statistics for the overall sample. For health outcomes, most of them (74%) considered their health status as excellent, very good, or good, 15% were current smokers, and 28% reported being obese. The CES-D score’s overall average was 1.42 (less than the depression diagnosis score of 3). The overall percentage of respondents who were outright owners, mortgaged owners, and renters are 47%, 35%, and 18%, respectively. Across the sample, 57% were females and 79% were Whites. Most of the respondents (88%) were US born, married (69%), and in the labor force (35% working and 52% partly retired). Respondents had an average age of 66.27 years. The average county-level all-age poverty rate was 12.9%, and the median household income was $56 387. Though the overall county-level average unemployment rate was at 5.7%, there was a considerable variation across time reflecting the booms and busts in labor market conditions, particularly around the Great Recession years.

**Table 1.** Summary Statistics of Respondents in 1992–2016 Health and Retirement Study

		*SD*		
Variable	Mean	Overall	Between	Within
*Health outcomes*				
Self-reported good health (yes/no)	0.74	0.44	0.38	0.28
CES-D score (0–8)	1.42	1.93	1.73	1.20
Obesity (yes/no)	0.28	0.45	0.41	0.22
Current smoking (yes/no)	0.15	0.36	0.35	0.16
*Housing*				
House tenure status				
Outright owner	0.47	0.50	0.44	0.28
Mortgaged owner	0.35	0.48	0.41	0.28
Renter	0.18	0.39	0.40	0.16
The natural logarithm of HPI	4.74	0.30	0.27	0.22
*Demographics*				
Female	0.57	0.49	0.50	0.00
US born	0.88	0.32	0.34	0.00
Race				
White	0.79	0.41	0.44	0.00
Black	0.16	0.37	0.39	0.00
Others	0.05	0.23	0.27	0.00
Hispanics	0.09	0.29	0.32	0.00
Education				
Less than high school	0.20	0.40	0.42	0.00
High school	0.34	0.47	0.47	0.00
Some college	0.23	0.42	0.42	0.00
College and above	0.23	0.42	0.41	0.00
Marital status				
Married	0.69	0.46	0.44	0.21
Divorced	0.11	0.31	0.31	0.13
Windowed	0.17	0.37	0.33	0.20
Never married	0.03	0.18	0.21	0.05
Labor force participation				
Working	0.35	0.48	0.42	0.30
Partly retired	0.52	0.50	0.41	0.34
Disabled	0.03	0.16	0.15	0.11
Outside labor force	0.10	0.30	0.28	0.20
Age	66.27	11.14	11.13	5.28
*County-level economic and health variables*				
ln (county-level poverty rate)	2.56	0.44	0.42	0.16
ln (county-level household median income)	10.94	0.24	0.23	0.07
ln (county-level unemployment rate)	1.74	0.42	0.33	0.29
ln (county-level number of beds)	7.17	1.62	1.60	0.44
Number of respondents (*N*)	34 182			
Number of observations (*N* × *T*)	174 759			

*Notes:* HPI = Housing Price Index; CES-D = Center for Epidemiologic Studies—Depression scale. CES-D scores were not asked in the first wave, leading to a different number of observations (*N* × *T* = 156 280).

Sample baseline characteristics are summarized separately for outright owners, mortgaged owners, and renters in Table 2. Because housing tenure status changes over time, statistics in Table 2 were obtained based on respondents’ information when they first entered the survey. In general, individuals with different housing tenure statuses were similar in sex, US born, zip code–level HPI, and other county-level variables, including poverty rate, median income, number of hospital beds, and unemployment rates. There were also differences in respondents’ characteristics across the 3 groups. For example, outright owners were more likely to be White (83% for outright owners, 76% for mortgaged owners, and 52% for renters), on average 9 years older than mortgaged owners and 6 years older than renters. There are 71% of mortgaged owners in the labor force, which is higher than that of outright owners (38%) and renters (46%). Renters include a larger proportion of Hispanics and have worse health status in physical health, mental health, and health-related behaviors.

**Table 2.** Summary Statistics of Respondents by Housing Tenure Status

Variable	Outright Owner	Mortgaged Owner	Renter	Total
Health outcomes				
Self-reported good health (yes/no)	0.75	0.82	0.60	0.75
CES-D score (0–8)	1.40	1.29	2.21	1.56
Obesity	0.22	0.29	0.35	0.28
Current smoking (yes/no)	0.16	0.20	0.32	0.21
*Housing*				
The natural logarithm of HPI	4.49	4.60	4.69	4.58
*Demographics*				
Female	0.55	0.53	0.60	0.55
US born	0.90	0.88	0.80	0.87
Race				
White	0.83	0.76	0.52	0.73
Black	0.12	0.17	0.34	0.19
Others	0.05	0.08	0.13	0.08
Hispanics	0.09	0.09	0.17	0.11
Education				
Less than high school	0.24	0.12	0.30	0.21
High school	0.36	0.30	0.33	0.33
Some college	0.21	0.27	0.24	0.24
College and above	0.19	0.31	0.13	0.22
Marital status				
Married	0.76	0.84	0.49	0.73
Divorced	0.07	0.09	0.25	0.12
Widowed	0.14	0.04	0.15	0.10
Never married	0.03	0.03	0.11	0.05
Labor force participation				
Working	0.38	0.71	0.46	0.54
Partly retired	0.40	0.16	0.26	0.27
Disabled	0.02	0.02	0.09	0.04
Outside labor force	0.20	0.11	0.19	0.16
Age	64.32	55.24	58.38	59.17
*County-level economic and health variables*				
ln (county-level poverty rate)	2.58	2.53	2.69	2.59
ln (county-level household median income)	10.92	10.97	10.92	10.94
ln (county-level unemployment rate)	7.20	7.40	7.77	7.41
ln (county-level number of beds)	1.82	1.84	1.92	1.85
Number of respondents (*N*)	10 273	12 155	6446	28 874

*Notes:* CES-D = Center for Epidemiologic Studies—Depression scale; HPI = Housing Price Index. Given the housing tenure status is changing over time, statistics in this table are obtained based on respondents’ information when they first entered the survey.

Table 3 documents the main results. Increases in housing prices were significantly associated with better self-reported health for both mortgaged owners and renters; the difference between the 2 groups was not statistically significant (*p* = .573). Specifically, the coefficient on self-reported health for renters (mortgaged owners) was 0.0350 (0.0281), which implies that a 100% increase in HPI was associated with a 3.50 (2.81) percentage points (pp) higher likelihood of reporting excellent/very good/good health status. For outright owners, the association was not statistically significant (0.0211, *p* = .05), but the effect size on self-reported health status was reasonably comparable to that of mortgaged owners. Only renters saw significant reductions in CES-D scores related to increases in housing prices; a 100% increase in HPI led to 0.2166 decreases in CES-D scores, which is approximately a standard deviation reduction in HRS data. Regarding health behaviors, we found that the likelihood of obesity decreased significantly for both outright owners and renters by 0.0182 and 0.0285, respectively, associated with a 100% increase in HPI. Renters also saw a significant drop in the probability of smoking (−0.0303, *p* < .001). Results were fairly consistent when levels of HPI were used in the primary regression model (Supplementary Table 1).

**Table 3.** Estimates of Housing Prices on Health From a Within-Subject Fixed-Effects Model

Variable	Good Health (Yes/No)	CES-D Score (0–8)	Obesity (Yes/No)	Smoking (Yes/No)
House tenure status				
Outright owner (Reference)				
Mortgage owner	−0.0434 (0.0393)	0.0838 (0.1842)	−0.0661 (0.0347)*	0.0462 (0.0283)
Renter	−0.0777 (0.0585)	0.9901 (0.2762)***	0.0446 (0.0499)	0.1864 (0.0431)***
Outright owner × ln(HPI)	0.0211 (0.0108)*	−0.0274 (0.0489)	−0.0182 (0.0093)**	0.0095 (0.0069)
Mortgage owner × ln(HPI)	0.0281 (0.0108)***	−0.0396 (0.0501)	−0.0038 (0.0097)	−0.0003 (0.0071)
Renter × ln(HPI)	0.0350 (0.0132)***	−0.2369(0.0628)***	−0.0285 (0.0117)***	−0.0303 (0.0095)***
Marital status				
Married (Reference)				
Divorced	−0.0136 (0.0072)*	0.3369 (0.0366)***	−0.0067 (0.0062)	0.0071 (0.0052)
Widowed	0.0200 (0.0053)***	0.4945 (0.0268)***	−0.0229 (0.0044)***	0.0076 (0.0036)**
Never married	0.0377 (0.0174)**	0.3417 (0.0940)***	−0.0202 (0.0149)	−0.0207 (0.0137)
Labor force participation				
Working (Reference)				
(partly) Retired	−0.0345 (0.0033)***	0.1057 (0.0156)***	0.0042 (0.0030)	−0.0140 (0.0024)***
Disabled	−0.1411 (0.0087)***	0.4868 (0.0475)***	0.0152 (0.0065)**	−0.0175 (0.0057)***
Outside labor force	−0.0284 (0.0047)***	0.1930 (0.0234)***	0.0114 (0.0038)***	−0.0094 (0.0030)***
Age	−0.0092 (0.0080)	0.0538 (0.0349)	0.0235 (0.0059)***	−0.0111 (0.0049)**
Age squared	−0.0002 (0.0000)***	0.0008 (0.0001)***	−0.0002 (0.0000)***	0.0001 (0.0000)***
ln (county-level poverty rate)	0.0289 (0.0134)**	−0.0635 (0.0619)	−0.0066 (0.0115)	0.0133 (0.0089)
ln (county-level household median income)	−0.0260 (0.0308)	0.0699 (0.1404)	0.0106 (0.0261)	0.0258 (0.0192)
ln (county-level number of beds)	−0.0148 (0.0053)***	0.0458 (0.0225)**	0.0032 (0.0049)	−0.0006 (0.0039)
ln (county-level unemployment rate)	0.0085 (0.0063)	0.0648 (0.0279)**	−0.0094 (0.0052)*	−0.0027 (0.0037)
Constant	2.1116 (0.5373)***	−0.1662 (2.4486)	−0.7690 (0.4181)*	0.3386 (0.3338)
County fixed effects	Yes	Yes	Yes	Yes
Year fixed effects	Yes	Yes	Yes	Yes
State-specific linear trends	Yes	Yes	Yes	Yes
*N*	32 374	30 314	32 374	32 374
*N* × *T*	174 759	156 280	174 759	174 759

*Notes:* CES-D = Center for Epidemiologic Studies—Depression scale; HPI = Housing Price Index. In parentheses are standard errors clustered at the individual level. *N* × *T* represents the number of person-years.

***p < .01, **p < .05, *p < .1.

As previous research suggests that older American homeowners are reluctant to use housing wealth to support other nonhousing consumption, the wealth effect channel through which housing prices affect older adults’ health should be limited. We tested the mediation effect of wealth by re-estimating our primary models, additionally controlling for households’ housing and nonhousing wealth. As given in Supplementary Table 2, the estimates on all outcomes slightly attenuate for outright owners and mortgaged owners and remain remarkably similar for renters. These results corroborate previous evidence on older adults’ spending behaviors and suggest that the wealth effect does not account for much of the health impact of housing price fluctuations.

To explore the role of local amenities and infrastructure, we compared respondents with multiple properties to those with only a primary residence. The former might be less exposed to the amenities than the latter, who are more likely to continuously live in the primary residence. Our results show that those with multiple properties experienced fewer health benefits associated with increases in housing prices than those with only a primary residence (Panels A and B of Supplementary Table 3). Additionally, we also found that females—who generally spend more time at home than males—saw more health gains as housing prices increase (Panels C and D of Supplementary Table 3). This further supports the importance of local amenities as a mechanism in promoting health.

Sensitivity analyses yielded consistent results (Supplementary Table 4). Restricting analyses to nonmovers yielded consistent results except that the coefficients of HPI on self-reported health slightly attenuated for homeowners and became statistically insignificant. However, the effect size was still of public health importance. This rules out the possibility that the results are driven by reverse causality due to individuals’ migration behaviors corresponding to housing price dynamics. In addition, the FEIVs approach yielded slightly larger effect sizes and revealed positive health effects of rising house prices for all respondents, including outright owners. This addresses the reverse causality problem and controls current shocks that influence health outcomes but are not directly affected by the lagged HPI, such as unexpected income or accidents. Analyses controlling for zip code–level fixed effects also produced similar results with statistically significant findings on self-reported health status for outright owners (*p* = .045). Excluding county-level economic and health controls yielded slightly attenuated effects, consistent with prior literature (59–61). All of these results corroborate our main findings.

## Discussion

In this article, we investigated the impact of housing price dynamics over the last decades on US older adults’ physical health, mental health, and health-related behaviors. Using individual fixed-effects models, we found that increases in housing prices were associated with better physical health for all homeowners and more favorable health-related behaviors for outright owners. Home renters were more likely to report better physical health, more health-related behaviors, and reductions in depression scores when housing prices increase. Our findings suggest health outcomes are procyclical for both homeowners and renters when economic expansions are measured by housing prices; health improves (deteriorates) as housing prices increase (decrease) for both older American homeowners and renters.

Our results that increasing housing prices are beneficial for homeowners’ physical health are consistent with previous findings from the United Kingdom (3) and Australia (2) using a similar individual fixed-effects approach. Our results are also in line with other US studies that find detrimental health effects associated with plunges in housing prices during the Great Recession (10,25). However, our results contrast to previous studies that show no statistically significant improvement in homeowners’ physical health when housing prices increase (26,29). There are at least 2 reasons for the difference. First, our study includes all HRS respondents (10 273 outright owners and 12 155 mortgaged owners) interviewed from 1992 to 2016, instead of a small sample of 4207 homeowners born 1924–1960 and interviewed in 2006 in the work of Hamoudi and Dowd (29); the large, representative, and the longitudinal sample provides sufficient statistical power. Compared to Sung and Qiu (26) who focus on adults of all ages, most of our sample are middle-aged and older adults whose physical health is more sensitive to changes in the living environment. Second, different from these 2 articles, we used an individual fixed-effects model. This controls for all time-invariant unobserved confounding factors such as genetics that could distort the relationship between residential-area housing prices and personal health. Additionally, because the HRS includes detailed information on homeownership information, we do not need to impute the homeownership as done in the work of Sung and Qiu (26).

In terms of homeowners’ mental health and health-related behaviors, our results are relatively consistent with a UK study that found no significant psychological health effect (3). They are inconsistent with previous similar US studies (10,26–28) and a study in Australia (2) that show significantly improved mental health for homeowners; we also found beneficial effects on homeowners’ mental health, but they are not statistically significant. Most importantly, our study lends no support to the wealth effect mechanism through which fluctuations in housing prices affect older American homeowners’ health, which contradicts previous studies (2,26) supporting this wealth channel for adults of all ages. But it further confirms the previous evidence that older adults rarely use housing wealth to support their general nonhousing consumption (12,14,23). Thus, the health impacts of housing prices on homeowners are probably through other channels such as local area amenities and economic prospects.

We found a large and significant beneficial health effect of housing price increases for older renters. Although previous studies demonstrate detrimental mental health effects for adult renters of all ages (2,26), the negative impact on US renters’ mental health does not persist in the long run (26). But our finding that increases in housing prices led to fewer depressive symptoms for US renters is consistent with that of a UK study, which like our study does not support a pure wealth effect (4). We also found that rising housing prices were associated with a decreased probability of obesity and smoking. Why did renters experience health benefits related to increasing housing prices? A plausible explanation is that housing prices also reflect changes in local area amenities and socioeconomic development. These factors could contribute to renters’ health gains from a booming housing market. Admittedly, this mechanism works for both homeowners and renters but apparently is greater for renters. First, rising housing prices could facilitate investment in the local living environment (eg, green space, crime, and commuting time to central business districts), which have considerable health implications for vulnerable residents (30,31,62). For example, the amount of green space in a neighborhood has significant effects on individuals’ perceived general health and mental health, with a stronger relationship for lower socioeconomic groups (32,62). Second, the housing market also reflects the local economic development that benefits renters, such as employment and economic outputs. Many studies have shown that rising house prices lead to business investment, business expansion, and job creation (20,34), while a decline in aggregate housing prices leads to a decrease in gross domestic products (35). Notably, economic downturns hit renters’ health harder than that of homeowners, as the latter could use their housing wealth as a buffer against financial shocks. Although our individual fixed-effects model can adjust many local area amenities and socioeconomic conditions, it cannot account for all of the changes over the study period. Third, promising economic prospects might prompt renters to increase health-enhancing consumption and investment, based on the lifecycle consumption theory (19). It would then facilitate renters to pursue a healthy lifestyle and reduce the probability of obesity and smoking.

This study was subject to several limitations. The primary limitation of this study is that the sample comprises mostly middle-aged and older adults. Those who were renters at the later stage of life represent a particular socially disadvantaged group. Therefore, the results of this study may not apply to younger adults. However, it is still an interesting population for this study’s purposes. Middle-aged and older adults were more likely to be homeowners (82% in our sample) and, therefore, more directly affected by housing price dynamics. The older adult population is also more vulnerable to changes in wealth and local socioeconomic conditions captured by housing prices. Second, although the fixed-effects model can control a wide range of time-constant confounding factors, we could not wholly control all time-varying confounding factors. As such, our estimates only indicate correlation, not causation. Third, recall bias was also possible in the HRS survey data set. Finally, attrition could be a potential concern in panel studies; for example, if sicker people were more likely to drop out of the survey, our estimates would be biased downward (63,64).

## Conclusion and Policy Implications

This study complements prior work on housing price movements and health by looking at the health impacts for US middle-aged and older adults with a fixed-effects model. Our results show beneficial health outcomes for homeowners and renters when housing prices increase, especially for renters. As we ruled out the wealth effect as a potential channel, the health impacts are more likely to be driven by changes in local amenities and economic development. Given that the correlation between housing price dynamics and health is stronger for individuals (eg, renters) from low socioeconomic groups, policy levers could provide more resources to those vulnerable populations during economic downturns. The explicit mechanism running from housing price dynamics to health for house renters warrants further research to allow us to propose more effective policy tools.

## Supplementary Material

igab012_suppl_Supplementary_Materials
