# Supplementary material for: Booms and Busts in Housing Market and Health Outcomes for Older Americans
Source: Innov Aging. 2021 Apr 10;5(2):igab012. doi: 10.1093/geroni/igab012 (PMC8152867; doi:10.1093/geroni/igab012)
Supplement: igab012_suppl_Supplementary_Materials [file igab012_suppl_supplementary_materials.docx]

**Online Supplementary Material for Publication in *Innovation in Aging*:**

**Booms and Busts in Housing Market and Health Outcomes for Older Americans**

Dahai Yue, PhD^1,*^ & Ninez A. Ponce, PhD^2^

1. Department of Health Policy and Management, University of Maryland, College Park, MD, USA.

2. Department of Health Policy and Management, University of California, Los Angeles, USA.

*Address correspondence to: Dahai Yue, PhD, 4200 Valley Drive, College Park, MD 20742. E-mail: dhyue@umd.edu

**Supplementary Table 1.** Full results from the primary fixed effects model using levels of HPI

**Supplementary Table 2.** Estimates of housing prices on health from a within-subject fixed effects model, controlling for housing and non-housing wealth

**Supplementary Table 3.** Estimates of housing prices on health by subgroups

**Supplementary Table 4.** Robustness checks of results from the main model

**Supplementary Figure 1.** Distribution of log-transformed Housing Price Index across years

**Supplementary Figure 2.** Sample flowchart

**Supplementary Figure 3.** Temporal trends of the median house price index

**Supplementary Table 1.** Full results from the primary fixed effects model using levels of HPI

| Variable | Good Health (yes/no) | CES-D Score  (0-8) | Obesity  (yes/no) | Smoking  (yes/no) |
| --- | --- | --- | --- | --- |
| House tenure status |  |  |  |  |
| Outright owner (Reference) |  |  |  |  |
| Mortgage owner | -0.0177** | 0.0419 | -0.0131* | 0.0076 |
|  | (0.0084) | (0.0396) | (0.0074) | (0.0059) |
| Renter | -0.0283** | 0.1880*** | 0.0039 | 0.0322*** |
|  | (0.0129) | (0.0608) | (0.0108) | (0.0093) |
| Outright owner * HPI | 0.0001* | -0.0001 | -0.0001** | 0.0001 |
|  | (0.0001) | (0.0003) | (0.0001) | (0.0000) |
| Mortgage owner * HPI | 0.0002*** | -0.0002 | -0.0000 | 0.0000 |
|  | (0.0001) | (0.0003) | (0.0001) | (0.0000) |
| Renter * HPI | 0.0003*** | -0.0017*** | -0.0002** | -0.0002*** |
|  | (0.0001) | (0.0004) | (0.0001) | (0.0001) |
| Marital status |  |  |  |  |
| Married (Reference) |  |  |  |  |
| Divorced | -0.0136* | 0.3371*** | -0.0067 | 0.0071 |
|  | (0.0072) | (0.0366) | (0.0062) | (0.0052) |
| Widowed | 0.0200*** | 0.4945*** | -0.0229*** | 0.0076** |
|  | (0.0053) | (0.0268) | (0.0044) | (0.0036) |
| Never married | 0.0377** | 0.3419*** | -0.0202 | -0.0208 |
|  | (0.0174) | (0.0940) | (0.0149) | (0.0137) |
| Labor force participation |  |  |  |  |
| Working (Reference) |  |  |  |  |
| (partly) Retired | -0.0345*** | 0.1058*** | 0.0042 | -0.0139*** |
|  | (0.0033) | (0.0156) | (0.0030) | (0.0024) |
| Disabled | -0.1410*** | 0.4871*** | 0.0152** | -0.0174*** |
|  | (0.0087) | (0.0475) | (0.0065) | (0.0057) |
| Outside labor force | -0.0284*** | 0.1932*** | 0.0114*** | -0.0094*** |
|  | (0.0047) | (0.0234) | (0.0038) | (0.0030) |
| Age | -0.0089 | 0.0530 | 0.0231*** | -0.0109** |
|  | (0.0080) | (0.0349) | (0.0059) | (0.0049) |
| Age Squared | -0.0002*** | 0.0008*** | -0.0002*** | 0.0001*** |
|  | (0.0000) | (0.0001) | (0.000) | (0.0000) |
| ln (county-level poverty rate) | 0.0294** | -0.0615 | -0.0066 | 0.0136 |
|  | (0.0134) | (0.0619) | (0.0115) | (0.0089) |
| ln (county-level household median income) | -0.0247 | 0.0604 | 0.0097 | 0.0258 |
|  | (0.0306) | (0.1399) | (0.0259) | (0.0191) |
| ln (county-level number of beds) | -0.0147*** | 0.0456** | 0.0032 | -0.0007 |
|  | (0.0053) | (0.0225) | (0.0049) | (0.0039) |
| ln (county-level unemployment rate) | 0.0094 | 0.0638** | -0.0098* | -0.0026 |
|  | (0.0064) | (0.0281) | (0.0052) | (0.0037) |
| Constant | 2.1567*** | -0.1359 | -0.8025* | 0.3642 |
|  | (0.5406) | (2.4610) | (0.4208) | (0.3357) |
| County fixed effects | Yes | Yes | Yes | Yes |
| Year fixed effects | Yes | Yes | Yes | Yes |
| State specific linear trends | Yes | Yes | Yes | Yes |
| Observations (N*T) | 174,759 | 156,280 | 174,759 | 174,759 |

*Notes.* CES-D = Center for Epidemiologic Studies Depression Scale. HPI = Housping Price Index. In parentheses are standard errors clustered at the individual level. N*T represents the number of person-years. *** p<0.01, ** p<0.05, * p<0.1.

**Supplementary Table 2.** Estimates of housing prices on health from a within-subject fixed effects model**,** controlling for housing and non-housing wealth

| Variable | Good Health (yes/no) | CES-D Score (0-8) | Obesity  (yes/no) | Smoking (yes/no) |
| --- | --- | --- | --- | --- |
| House tenure status |  |  |  |  |
| Outright owner (Reference) |  |  |  |  |
| Mortgage owner | -0.0430 | 0.0811 | -0.0665* | 0.0459 |
|  | (0.0392) | (0.1842) | (0.0347) | (0.0283) |
| Renter | -0.0797 | 0.9990*** | 0.0459 | 0.1862*** |
|  | (0.0585) | (0.2765) | (0.0499) | (0.0431) |
| Outright owner * ln(HPI) | 0.0206* | -0.0251 | -0.0179* | 0.0096 |
|  | (0.0108) | (0.0490) | (0.0093) | (0.0069) |
| Mortgage owner * ln(HPI) | 0.0275** | -0.0367 | -0.0034 | -0.0002 |
|  | (0.0109) | (0.0502) | (0.0097) | (0.0071) |
| Renter * ln(HPI) | 0.0350*** | -0.2369*** | -0.0285** | -0.0302*** |
|  | (0.0132) | (0.0628) | (0.0117) | (0.0095) |
| Marital status |  |  |  |  |
| Married (Reference) |  |  |  |  |
| Divorced | -0.0134* | 0.3360*** | -0.0069 | 0.0072 |
|  | (0.0072) | (0.0366) | (0.0062) | (0.0052) |
| Widowed | 0.0202*** | 0.4939*** | -0.0230*** | 0.0076** |
|  | (0.0053) | (0.0268) | (0.0044) | (0.0036) |
| Never married | 0.0379** | 0.3410*** | -0.0204 | -0.0206 |
|  | (0.0174) | (0.0940) | (0.0149) | (0.0137) |
| Labor force participation |  |  |  |  |
| Working (Reference) |  |  |  |  |
| (partly) Retired | -0.0346*** | 0.1057*** | 0.0042 | -0.0140*** |
|  | (0.0033) | (0.0156) | (0.0030) | (0.0024) |
| Disabled | -0.1410*** | 0.4867*** | 0.0152** | -0.0175*** |
|  | (0.0087) | (0.0475) | (0.0065) | (0.0057) |
| Outside labor force | -0.0284*** | 0.1930*** | 0.0114*** | -0.0094*** |
|  | (0.0047) | (0.0234) | (0.0038) | (0.0030) |
| Age | -0.0093 | 0.0542 | 0.0236*** | -0.0111** |
|  | (0.0080) | (0.0349) | (0.0059) | (0.0049) |
| Age Squared | -0.0002*** | 0.0008*** | -0.0002*** | 0.0001*** |
|  | (0.0000) | (0.0001) | (0.0000) | (0.0000) |
| ln (county-level poverty rate) | 0.0289** | -0.0635 | -0.0066 | 0.0132 |
|  | (0.0134) | (0.0619) | (0.0115) | (0.0089) |
| ln (county-level household median income) | -0.0264 | 0.0712 | 0.0108 | 0.0255 |
|  | (0.0308) | (0.1404) | (0.0261) | (0.0191) |
| ln (county-level number of beds) | -0.0148*** | 0.0458** | 0.0032 | -0.0006 |
|  | (0.0053) | (0.0225) | (0.0049) | (0.0039) |
| ln (county-level unemployment rate) | 0.0085 | 0.0648** | -0.0094* | -0.0027 |
|  | (0.0063) | (0.0279) | (0.0052) | (0.0037) |
| Non-housing wealth (million) | 0.0011 | -0.0026 | -0.0006 | 0.0013*** |
|  | (0.0008) | (0.0031) | (0.0006) | (0.0005) |
| Housing wealth (million) | 0.0021 | -0.0101 | -0.0015 | -0.0007 |
|  | (0.0021) | (0.0094) | (0.0018) | (0.0010) |
| Constant | 2.1218*** | -0.2044 | -0.7752* | 0.3439 |
|  | (0.5373) | (2.4483) | (0.4182) | (0.3338) |
| N | 32,374 | 30,314 | 32,374 | 32,374 |
| N*T | 174,759 | 156,280 | 174,759 | 174,759 |

*Notes*. CES-D = Center for Epidemiologic Studies Depression Scale. HPI = Housping Price Index. In parentheses are standard errors clustered at the individual level. N*T represents the number of person-years. *** p<0.01, ** p<0.05, * p<0.1.

**Supplementary Table 3.** Estimates of housing prices on health by subgroups

| Variable | Good health (yes/no) | CES-D score (0-8) | Obesity  (yes/no) | Smoking (yes/no) |
| --- | --- | --- | --- | --- |
| *Panel A. Homeowners with housing wealth outside of the primary residence* | | | | |
| Outright Owner * ln(HPI) | 0.0380 | 0.0382 | -0.0815 | 0.0205 |
|  | (0.0281) | (0.1298) | (0.2010) | (0.0243) |
| Mortgaged Owner * ln(HPI) | 0.0255 | 0.0094 | 0.0365 | 0.0275 |
|  | (0.0286) | (0.1290) | (0.2116) | (0.0263) |
| N | 8,966 | 8,193 | 8,966 | 8,966 |
| N*T | 27,297 | 23,852 | 27,297 | 27,297 |
| *Panel B. Homeowners with only a primary residence* | | | | |
| Outright Owner * ln(HPI) | 0.0122 | -0.1204** | -0.1698* | -0.0248** |
|  | (0.0129) | (0.0587) | (0.0992) | (0.0112) |
| Mortgaged Owner * ln(HPI) | 0.0146 | -0.1201** | -0.1180 | -0.0153 |
|  | (0.0131) | (0.0607) | (0.1030) | (0.0116) |
| N | 25,397 | 23,962 | 25,397 | 25,397 |
| N*T | 115,821 | 103,812 | 115,821 | 115,821 |
| *Panel C. Females* |  |  |  |  |
| Outright Owner * ln(HPI) | 0.0194 | -0.0399 | -0.1673 | -0.0303** |
|  | (0.0142) | (0.0680) | (0.1196) | (0.0123) |
| Mortgaged Owner * ln(HPI) | 0.0299** | -0.0220 | -0.0635 | -0.0197 |
|  | (0.0142) | (0.0696) | (0.1249) | (0.0131) |
| Renter * ln(HPI) | 0.0300* | -0.2136** | -0.6004*** | -0.0506*** |
|  |  |  |  |  |
| N | 19,070 | 18,383 | 19,070 | 19,070 |
| N*T | 100,083 | 91,898 | 100,083 | 100,083 |
| *Panel D. Males* |  |  |  |  |
| Outright Owner * ln(HPI) | 0.0246 | -0.0137 | -0.1883* | -0.0016 |
|  | (0.0166) | (0.0681) | (0.1101) | (0.0141) |
| Mortgaged Owner * ln(HPI) | 0.0264 | -0.0649 | -0.0322 | 0.0177 |
|  | (0.0169) | (0.0700) | (0.1139) | (0.0146) |
| Renter * ln(HPI) | 0.0370* | -0.2519*** | -0.1650 | 0.0079 |
|  |  |  |  |  |
| N | 15,112 | 14,065 | 15,112 | 15,112 |
| N*T | 74,676 | 64,382 | 74,676 | 74,676 |

*Notes.* CES-D = Center for Epidemiologic Studies Depression Scale. HPI = Housping Price Index. In parentheses are standard errors clustered at individual levels. N denotes the number of persons, N*T represents the number of person-years.

*** p<0.01, ** p<0.05, * p<0.1.

**Supplementary Table 4.** Robustness checks of results from the main model

|  | Good health (yes/no) | CES-D score (0-8) | Obesity  (yes/no) | Smoking (yes/no) |
| --- | --- | --- | --- | --- |
| *Panel A. Restrict to non-movers* |  |  |  |  |
| Outright Owner * ln(HPI) | 0.0153 | -0.0418 | -0.0131 | -0.0021 |
|  | (0.0127) | (0.0576) | (0.0109) | (0.0079) |
| Mortgaged Owner * ln(HPI) | 0.0170 | -0.0418 | -0.0001 | -0.0128* |
|  | (0.0130) | (0.0592) | (0.0115) | (0.0081) |
| Renter * ln(HPI) | 0.0483*** | -0.3709*** | -0.0539*** | -0.0470*** |
|  | (0.0176) | (0.0807) | (0.0148) | (0.0132) |
| N | 32,374 | 30,314 | 32,374 | 32,374 |
| N*T | 138,029 | 124,230 | 138,029 | 138,029 |
| *Panel B. Fixed effects instrumental variables* |  |  |  |  |
| Outright Owner * ln(HPI) | 0.0205** | -0.0223 | -0.0175** | 0.0091 |
|  | (0.0103) | (0.0458) | (0.0082) | (0.0055) |
| Mortgaged Owner * ln(HPI) | 0.0286*** | -0.0290 | 0.0042 | -0.0052 |
|  | (0.0105) | (0.0469) | (0.0084) | (0.0057) |
| Renter * ln(HPI) | 0.0454*** | -0.2742*** | -0.0250** | -0.0276*** |
|  | (0.0122) | (0.0543) | (0.0097) | (0.0066) |
| N | 28,403 | 27,331 | 28,406 | 28,393 |
| N*T | 147,069 | 137,910 | 147,160 | 146,278 |
| *Panel C. Zip-code fixed effects* | | | | |
| Outright Owner * ln(HPI) | 0.0220** | -0.0178 | -0.0158 | 0.0033 |
|  | (0.0112) | (0.0505) | (0.0096) | (0.0070) |
| Mortgaged Owner * ln(HPI) | 0.0238** | -0.0324 | -0.0043 | -0.0075 |
|  | (0.0114) | (0.0515) | (0.0101) | (0.0072) |
| Renter * ln(HPI) | 0.0488*** | -0.2877*** | -0.0324*** | -0.0359*** |
|  | (0.0143) | (0.0667) | (0.0122) | (0.0103) |
| N | 34,182 | 32,799 | 34,182 | 34,182 |
| N*T | 174,759 | 159,797 | 174,759 | 174,759 |
| *Panel D. Excluding economic and health controls* | | | | |
| Outright Owner * ln(HPI) | 0.0093 | -0.0220 | -0.0130 | 0.0108* |
|  | (0.0100) | (0.0456) | (0.0085) | (0.0064) |
| Mortgaged Owner * ln(HPI) | 0.0157 | -0.0353 | 0.0018 | 0.0010 |
|  | (0.0099) | (0.0464) | (0.0089) | (0.0065) |
| Renter * ln(HPI) | 0.0229* | -0.2341*** | -0.0230** | -0.0293*** |
|  | (0.0126) | (0.0600) | (0.0111) | (0.0092) |
| N | 32,374 | 30,314 | 32,374 | 32,374 |
| N*T | 174,759 | 156,280 | 174,759 | 174,759 |

*Notes.* CES-D = Center for Epidemiologic Studies Depression Scale. HPI = Housping Price Index.

Panel A restricted to person-year observations for those in the analytic sample who remained at the home address since they first entered the HRS survey.

Panel B used two-year (or one-wave) lagged log transformed HPI as instrumental variables.

Panel C included zip-code fixed effects. In parentheses are standard errors clustered at individual levels.

Panel D excluded all county-level economic and health controls.

N denotes the number of persons, N*T represents the number of person-years.

*** p<0.01, ** p<0.05, * p<0.1.

**Supplementary Figure 1.** Distribution of log-transformed Housing Price Index across years. *Notes*. 2000 is the base year for the Housing Price Index (HPI) with HPI=100.

**Supplementary Figure 2.** Sample flowchart

**Supplementary Figure 3:** Temporal trends of the median house price index. *Notes.* FHFA HPI represents the median value of the Housing Price Index calculated based on the Federal Housing Finance Agency database, HRS HPI denotes the median HPI using the HRS full sample, and Sample HPI indicates the median HPI based on the HRS analytic sample.
